# 

**Published:** 1913-09-13

**Authors:** 


### Appointments.

The recently appointed resident medical officers at the Coventry and Warwickshire Hospital, who began their duties this month, are: Dr. H. M. Anderson, M.B., Ch.B., Edin., senior house surgeon, late junior house surgeon and senior house surgeon, Royal Infirmary, Edinburgh; senior house surgeon and surgeon registrar, Royal Hospital, Portsmouth; medical and surgical officer, Royal Hospital, Preston; previously deputy medical superintendent, Forest Gate Infirmary, London. Dr. Evan H. Jones, M.B., B.S., London, M.R.C.S., L.R.C.P., junior house surgeon, late junior house surgeon, Royal Infirmary, Preston, and resident medical and surgical officer, Royal Infirmary, Preston; Dr. Jones has also held an appointment at Forest Gate Infirmary. Dr. Norman B. Graham, B.A., M.B., B.Ch., B.A.O., Queen's University, Belfast, house physician, at present assistant medical officer at Herts County Mental Hospital, St. Albans.

### ACCIDENT TO A LONDON SURGEON.

Mr. Albert Boyce Barrow, F.R.C.S., surgeon to King's College Hospital, was injured last week in a motor accident at Inworth, Essex. While on his way home from a day's partridge shooting, according to accounts, his motor-car ran into a ditch. Its window was smashed and Mr. Barrow received two cuts on the head. The cause of the accident has not been published, but at the time of writing Mr. Barrow is reported to be making satisfactory progress.

